# Supplementary material for: Costless metabolic secretions as drivers of interspecies interactions in microbial ecosystems
Source: Nat Commun. 2019 Jan 9;10:103. doi: 10.1038/s41467-018-07946-9 (PMC6327061; doi:10.1038/s41467-018-07946-9)
Supplement: Supplementary file 1 — Supplementary Information [file 41467_2018_7946_MOESM1_ESM.pdf]

Costless metabolic secretions as drivers of interspecies interactions in microbial ecosystems

Pacheco *et al*.

SUPPLEMENTARY INFORMATION

SUPPLEMENTARY FIGURES

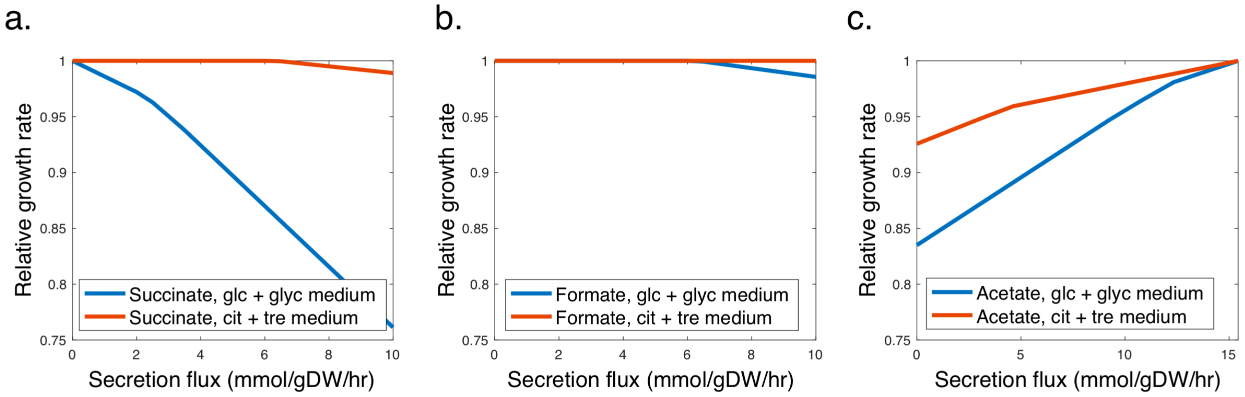

Supplementary Figure 1 Three modes of *in silico* metabolite secretion by *E. coli* (iJO1366) in anoxic conditions as defined by FBA. What makes a metabolite costless is dependent on the environment. **a** Increasing the secretion flux of a 'costly' product, such as succinate, imposes a reduction in growth rate when glucose and glycerol are supplied as carbon sources. When the carbon sources are replaced with citrate and trehalose, succinate is secreted without a cost to growth rate. **b** With glucose and glycerol as carbon sources, *E. coli* is predicted to have a wide range of fluxes at which formate can be secreted without a cost to its growth rate. Formate would, according to our definition, be secreted 'costlessly' by *E. coli* under the applied environmental conditions. **c** Some costlessly-secreted metabolites must be secreted at a given rate in order to maximize growth. If an upper bound is placed on acetate secretion, *E. coli* must allocate resources away from biomass in order to cope with its limited ability to secrete fermentation byproducts. Acetate would therefore also be considered a costlessly-secreted metabolite by our definition.

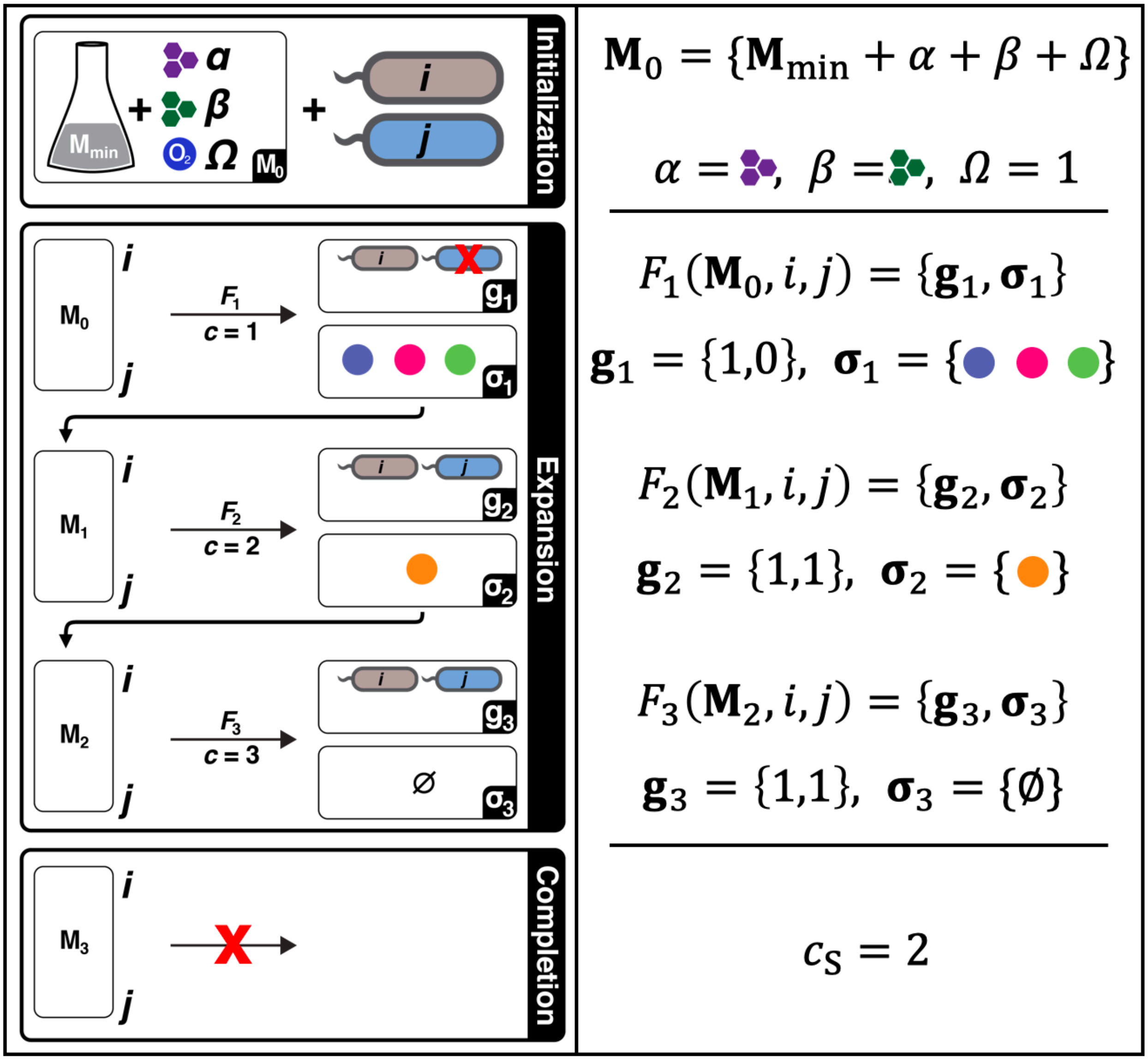

Supplementary Figure 2 Detailed example of single *in silico* experiment, illustrating three phases. Initialization: A minimal medium $\mathbf{M}_{\min}$ common to all simulated conditions (composed of salts, metals, vitamins, as well as nitrogen, phosphorous, and sulphur sources) is defined prior to execution of the pipeline. This medium is supplemented with two carbon sources, α and β.The Boolean variable $\Omega = \{0,1\}$ defines whether or not oxygen is present in the environment. Here, $\Omega = 1$. These together define the initial medium set, $\mathbf{M}_0$. Expansion: The function F is applied to genome-scale metabolic models of two organisms (i, j) in a series of iterations, c. In each iteration, F simulates the growth of both organisms in the current medium condition and returns the Boolean growth statuses $\mathbf{g}_c = \{g_i, g_j\}$ of both organisms and the set of any costlessly-secreted metabolites, $\boldsymbol{\sigma}_c$. Here, in the first iteration, $\boldsymbol{g}_1 = \{1,0\}$ since organism i grew but organism j did not. Since at least one organism in the pair grew, the medium is updated ($\mathbf{M}_{c+1} = \mathbf{M}_c + \boldsymbol{\sigma}_c$) and F is applied again until no new metabolites are secreted. Completion: When no new metabolites are added to the medium, the experiment is complete. The last iteration with any new secreted metabolites is defined as c_S.

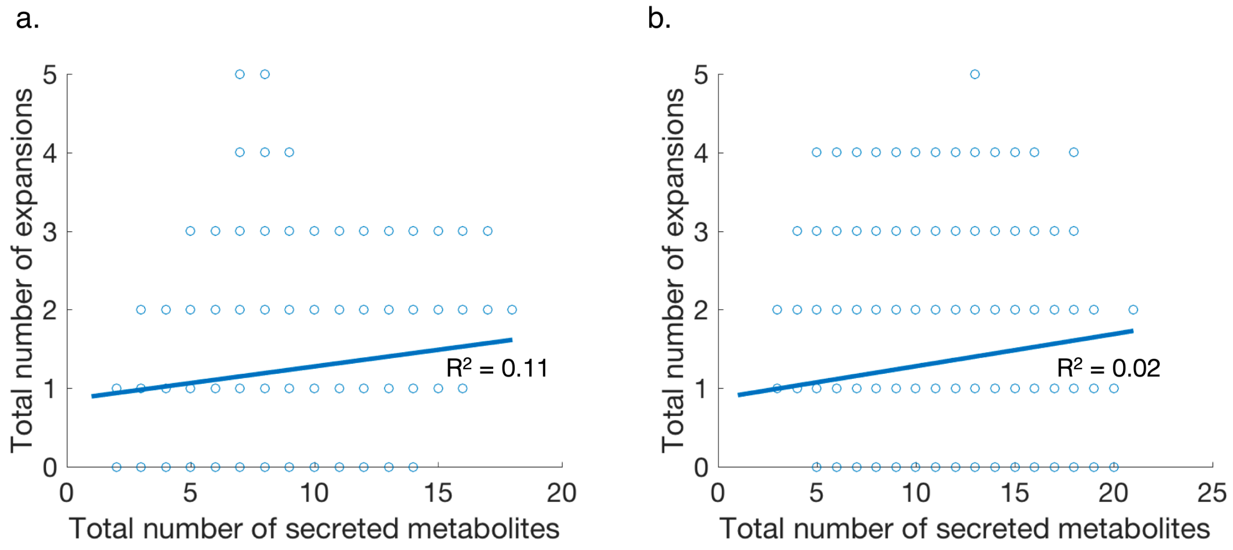

Supplementary Figure 3 Correlation between total number of metabolites secreted costlessly and the number of expansions in each *in silico* experiment. **a**,**b** correlation for simulations with (**a**) and without (**b**) oxygen. We observe a poor correlation between number of secreted metabolites and number of expansions in both oxic and anoxic simulations. This lack of correlation suggests a lower rate of metabolite exchange with increasing iterations, with most organisms quickly stabilizing their environment within one or two expansions. With oxygen, for example, only the *K. pneumoniae* and *Synechocystis* pair exhibited more than three medium expansions, with acetate, formate, citrate, and L-malate being the only metabolites secreted at these iterations. These scenarios accounted for only 40 simulations. Without oxygen, there were 697 simulations that reached more than three medium expansions, with 10 organisms being represented. However, this anaerobic set was dominated by the *S. cerevisiae*-*P. aeruginosa* pair, with fermentation byproducts being secreted at late iterations.

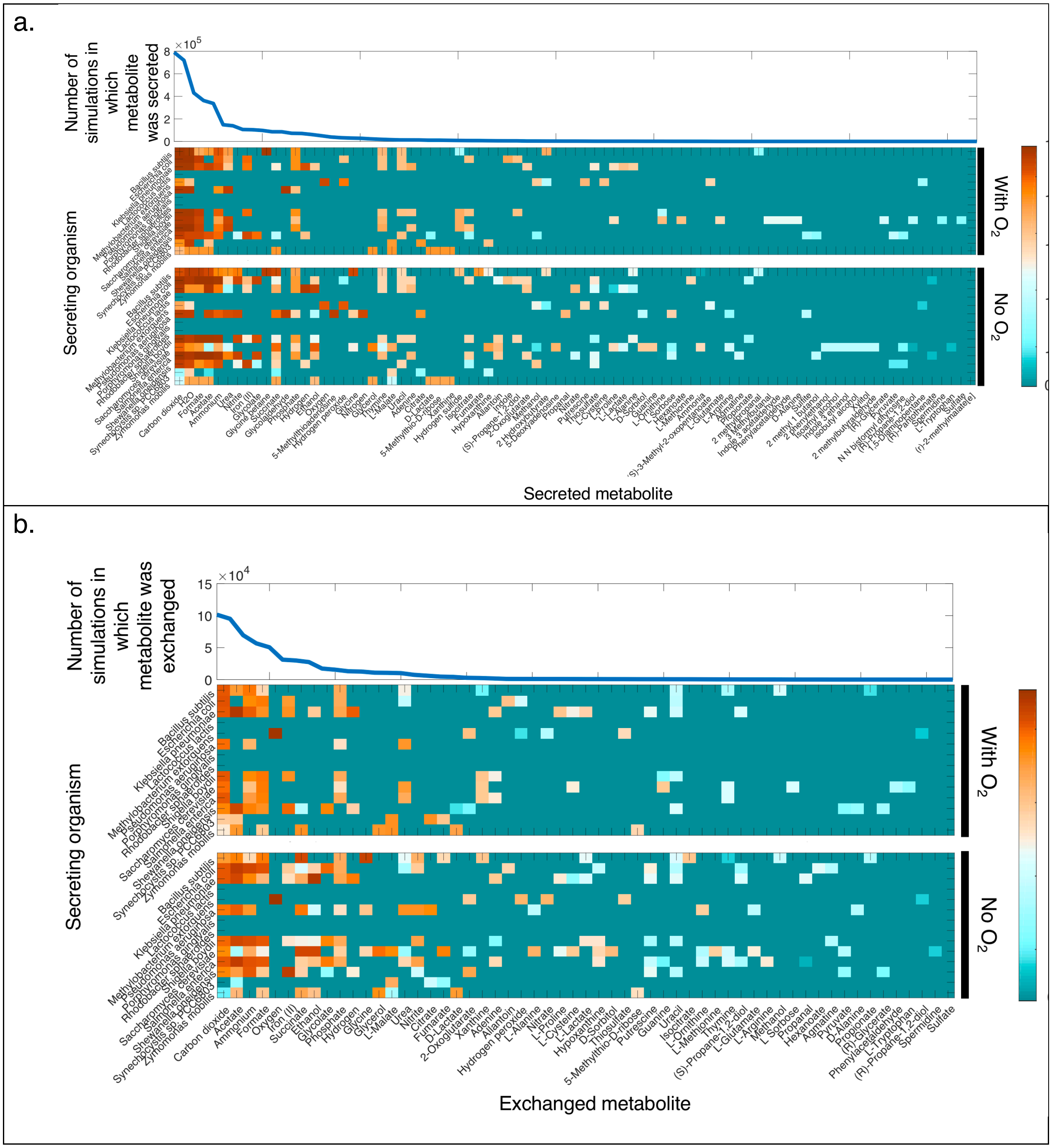

Supplementary Figure 4 Range of costlessly-secreted and exchanged metabolites. **a** Cumulative sum of *in silico* experiments in which metabolite was secreted (top), and sorted heatmap of metabolites secreted in at least one simulation, arranged by secreting organism (bottom). **b** Cumulative sum of *in silico* experiments in which each secreted metabolite was taken up by another organism (top), and sorted heatmap of metabolites secreted and taken up in at least one simulation, arranged by secreting organism (bottom).

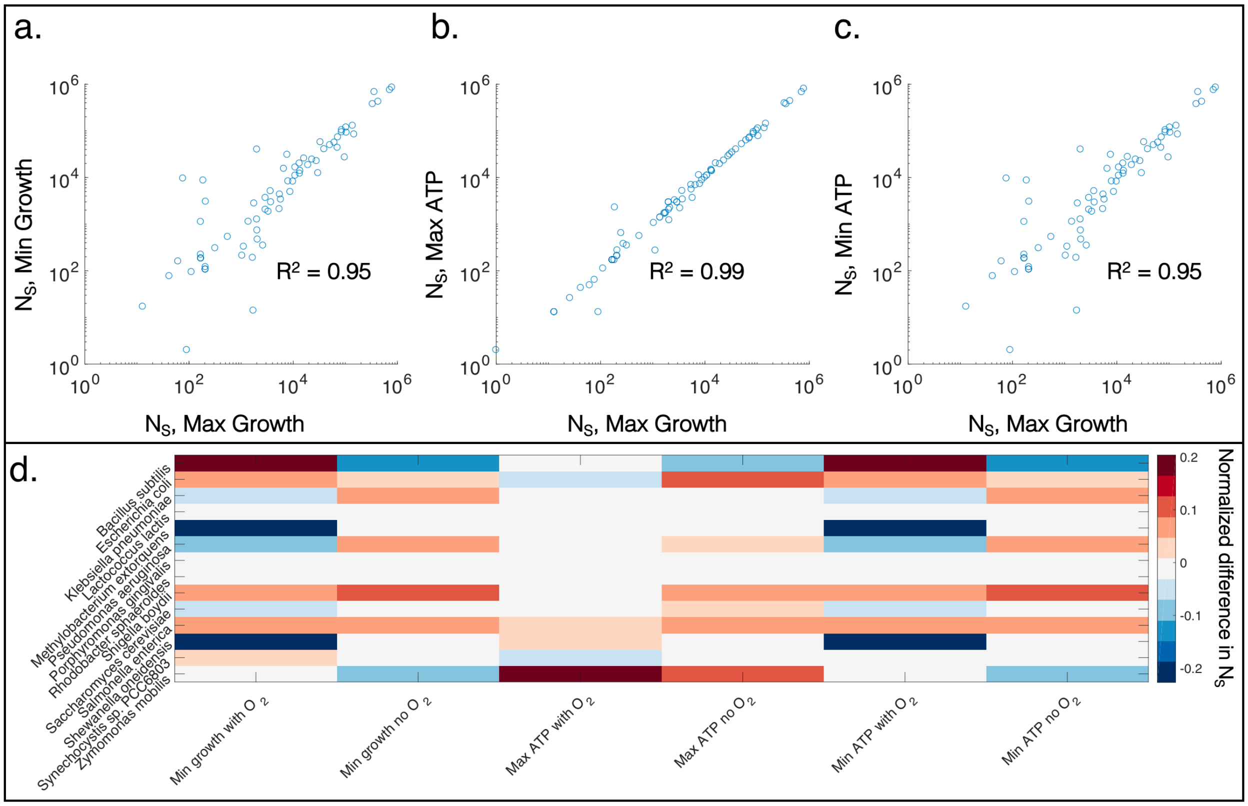

Supplementary Figure 5 Comparison of secretion profiles under alternative objective functions. The three alternative objectives (minimization of growth "Min Growth," maximization of ATP production "Max ATP," and minimization of ATP production "Min ATP") are compared to the growth maximization "Max Growth" objective. N_S is defined as the number of simulations in which a metabolite was secreted. **a**,**b**,**c** Comparison of values of N_S between Max Growth and Min Growth (**a**), Max ATP (**b**), and Min ATP (**c**), respectively. Secretion profiles predicted under Max Growth were robust, with metabolite secretion frequencies correlating highly between it and Min Growth ($R^2 = 0.95$), Max ATP ($R^2 = 0.99$), and Min ATP ($R^2 = 0.95$). The most similar condition to Max Growth was Max ATP, with only one metabolite (5'-Deoxyadenosine) being reported under Max Growth and not under Max ATP. We observed greater differences in predicted secretions between the maximization and minimization objectives, with 9 metabolites reported under Max Growth and Max ATP that were not present under Min Growth or Min ATP (Supplementary Table 5). **d** Heatmap showing differences in N_S by organism ($\mu = 0.002 \pm 0.033$). Differences are normalized by the values of N_S under growth maximization for each organism.

a.

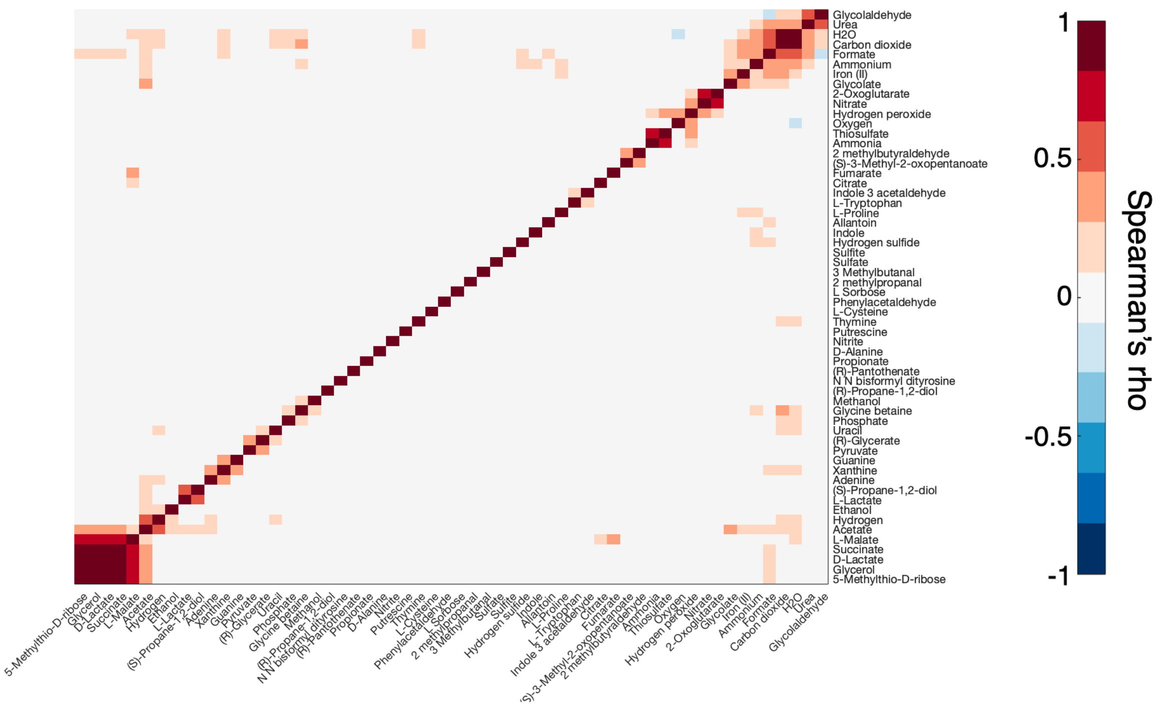

b.

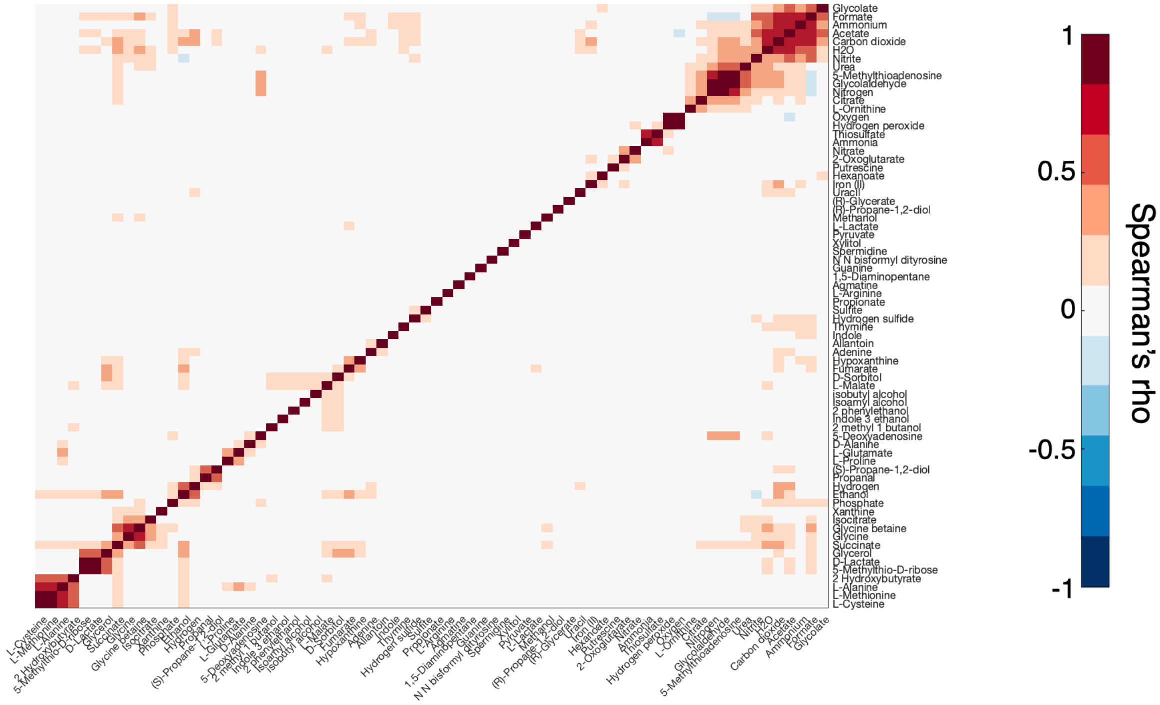

Supplementary Figure 6 Clustered Spearman correlation of secreted metabolites for simulation set. **a** Clustered correlations for simulations with oxygen. A strong co-occurrence of carbon-containing compounds (e.g. acetate, succinate, glycerol, lactate, malate) is observed. Positive correlations between these molecules and central carbon intermediates (e.g. citrate, fumarate, 2-oxoglutarate) are also present, in addition to co-secretion of nitrogen-containing compounds (e.g. ammonium, nitrate, urea). **b** Clustered correlations for simulations without oxygen. Strong correlations in secretion are observed between fermentation products and nitrogen-containing compounds, as well as among some amino acids (e.g. cysteine, methionine, alanine).

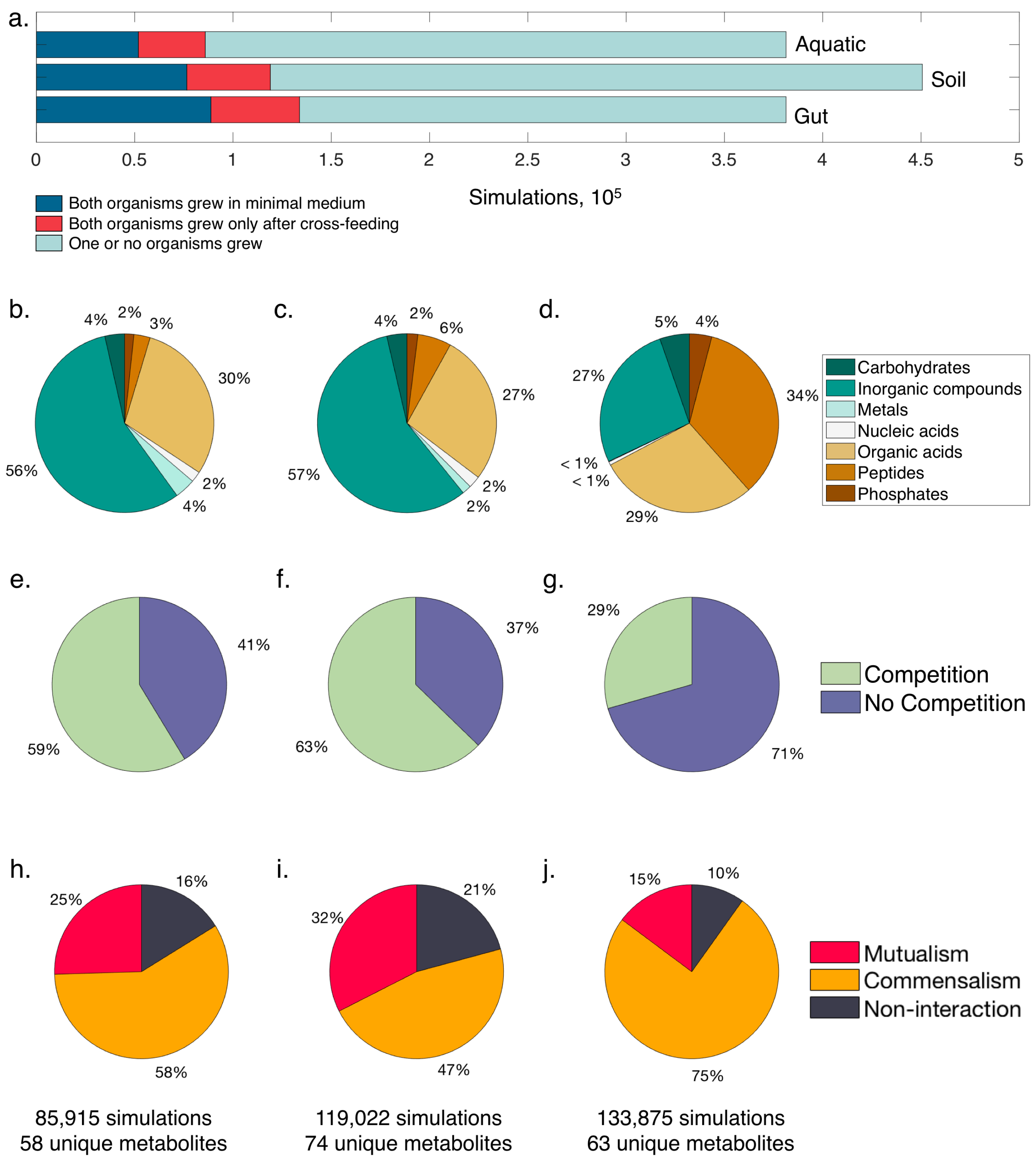

Supplementary Figure 7 Habitat-specific secretion patterns, growth outcomes, and interaction patterns. **a** Growth outcomes of all simulations, grouped by pairwise growth phenotype. Exchange of costlessly-secreted metabolic products can allow for substantial increases in the ability of organisms to survive (increases in growth-supporting environments of 65.5% in aquatic habitats, 55.5% in soil habitats, and 50.7% in gut habitats). **b-d** Categories of secreted metabolites for aquatic, soil, and gut-associated microbes respectively. Percentages are relative to the number of simulations in which both organisms grew. **e-f** Overall distributions of competitive/noncompetitive interactions for aquatic, soil, and gut-associated microbes respectively. **h-j** Overall distributions of general interactions mediated by costless metabolites for aquatic, soil, and gut-associated microbes respectively. These interactions at the level of secreted metabolites exist simultaneously with competition or no competition for a primary carbon source.

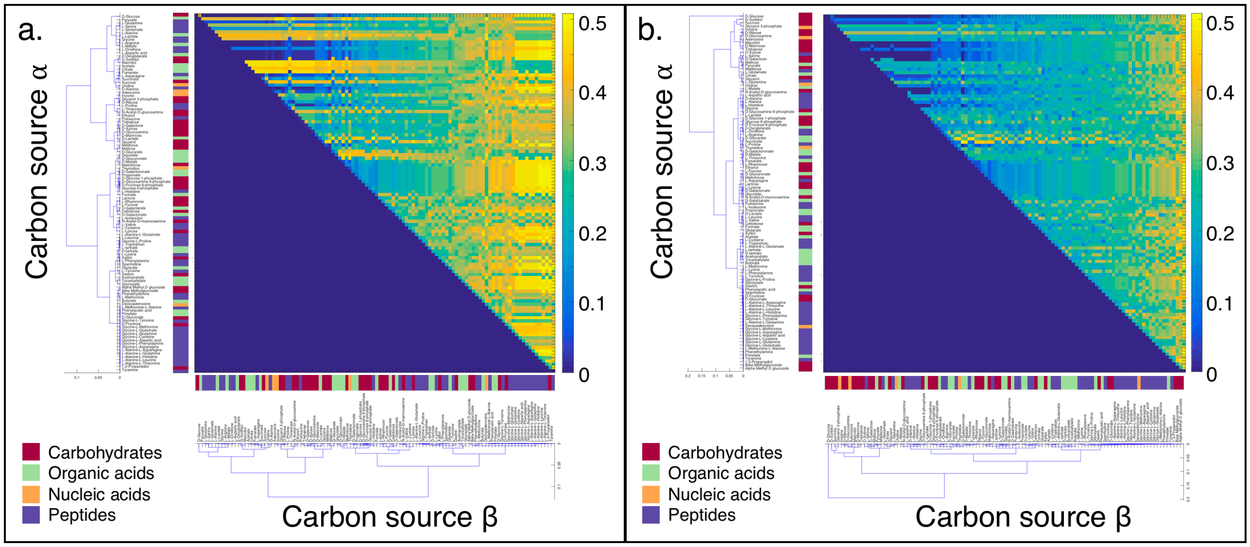

Supplementary Figure 8 Cooperativity indices of all carbon source pairs. **a, b** Cooperativity indices for simulations with (**a**) and without oxygen (**b**). Heatmaps are clustered by average carbon source cooperativity index. We find that simple sugars generally exhibit relatively low cooperativity indices, meaning that they are able to sustain growth efficiently on their own. More complex molecules and dipeptides exhibit higher average cooperativity indices, indicating they are more effective in allowing for organism growth when in the presence of another carbon source. Distinct clusters of carbohydrates and amino acids appear, suggesting carbon sources have similar cooperative effects by type. Carbon sources are listed in Supplementary Table 6 for enhanced visibility, in the order they are displayed here.

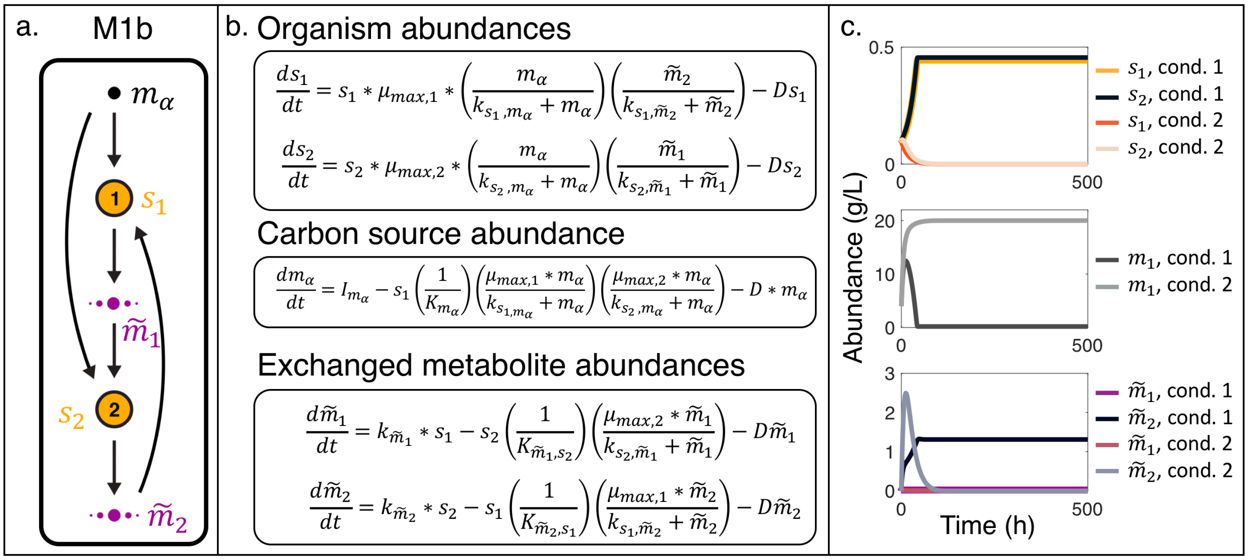

Supplementary Figure 9 Example of chemostat dynamical modeling for motif M1b (mutualism with one carbon source consumed and competition). **a** Schematic of motif, demonstrating all state variables and direction of metabolite flow. **b** Differential equations for modeling the motif. The organism abundances are defined by a maximum specific growth rate, $\mu_{\max}$, as well as the availabilities of the carbon sources and exchanged metabolites on which they depend. Carbon source abundances are defined by a constant influx rate, I_{m}, and by the consumption rate of each organism. Exchange metabolite abundances are defined by the abundance and secretion rate of the producing organism, as well as by the consumption rate of the consuming organism. All quantities are also governed by a dilution rate in a simulated chemostat. **c** Trajectories of state variables under two conditions, each defined by the maximum specific growth rates of the organisms. Condition 1: $\mu_{\max,1} = 0.25\ \mathrm{hr}^{-1}$, $\mu_{\max,2} = 0.8\ \mathrm{hr}^{-1}$; Condition 2: $\mu_{\max,1} = 0.15\ \mathrm{hr}^{-1}$, $\mu_{\max,2} = 0.8\ \mathrm{hr}^{-1}$. The dilution rate is set to $0.2\ \mathrm{hr}^{-1}$ in both conditions and the remaining parameters and initial conditions are defined in Supplementary Table 3. Condition 1 shows a stabilization of the system with both organisms reaching similar abundances at equilibrium. This occurs despite organism 2 having a much higher maximum specific growth rate than organism 1, as organism 2 must scale its effective growth rate down to account for its dependence on the secreted metabolite from organism 1 ($\widetilde{m}_1$). In condition 2, although the maximum specific growth rate of organism 2 is high, the maximum specific growth rate of organism 1 is less than the dilution rate. This difference leads to organism 1 being eliminated from the system, which, in turn, eliminates organism 2 due to its dependence on organism 1.

SUPPLEMENTARY TABLES

Supplementary Table 1: Carbon sources used in pairwise simulations. Metabolite names follow the naming convention used by the BIGG database [1], a collection of curated genome-scale metabolic models.

Carbon Source Name	BIGG Metabolite Name	Subcategory 2	Subcategory 1	Category
1,3-Propanediol	13ppd	Sugar alcohols	Monosaccharides	Carbohydrates
Acetate	ac	Monocarboxylic acids	Carboxylic acids	Organic acids
Acetoacetate	acac	3-Oxocarboxylic acids	Carboxylic acids	Organic acids
N-Acetyl-D-glucosamine	acgam	Amino sugars	Monosaccharides	Carbohydrates
N-Acetyl-D-mannosamine	acmana	Amino sugars	Monosaccharides	Carbohydrates
Adenosine	adn	Ribonucleosides	Nucleosides	Nucleic acids
2-Oxoglutarate	akg	2-Oxocarboxylic acids	Carboxylic acids	Organic acids
D-Alanine	ala-D	Other amino acids	Amino acids	Peptides
L-Alanine-L-Asparagine	ala-L-asp-L	Dipeptides	Amino acids	Peptides
L-Alanine-L-Glutamine	ala-L-gln-L	Dipeptides	Amino acids	Peptides
L-Alanine-L-Glutamate	ala-L-glu-L	Dipeptides	Amino acids	Peptides
L-Alanine-L-Histidine	ala-L-his-L	Dipeptides	Amino acids	Peptides
L-Alanine-L-Leucine	ala-L-leu-L	Dipeptides	Amino acids	Peptides
L-Alanine-L-Threonine	ala-L-thr-L	Dipeptides	Amino acids	Peptides
L-Alanine	ala-L	Common amino acids	Amino acids	Peptides
L-Arginine	arg-L	Common amino acids	Amino acids	Peptides
L-Asparagine	asn-L	Common amino acids	Amino acids	Peptides
L-Aspartic acid	asp-L	Common amino acids	Amino acids	Peptides
Butyrate	but	Monocarboxylic acids	Carboxylic acids	Organic acids
Cellobiose	cellb	Disaccharides	Disaccharides	Carbohydrates
Citrate	cit	Tricarboxylic acids	Carboxylic acids	Organic acids
L-Cysteine	cys-L	Common amino acids	Amino acids	Peptides
Deoxyadenosine	dad_2	Deoxyribonucleosides	Nucleosides	Nucleic acids
Dextrin	dextrin	Polysaccharides	Polysaccharides	Carbohydrates
Ethanol	etoh	Sugar alcohols	Monosaccharides	Carbohydrates
D-Fructose 6-phosphate	f6p	Sugar 6 phosphates	Sugar phosphates	Carbohydrates
Formate	for	Monocarboxylic acids	Carboxylic acids	Organic acids
Fructose	fru	Ketoses	Monosaccharides	Carbohydrates
L-Fucose	fuc-L	Deoxy sugars	Monosaccharides	Carbohydrates
Fumarate	fum	Dicarboxylic acids	Carboxylic acids	Organic acids
D-Glucose 1-phosphate	g1p	Sugar 1 phosphates	Sugar phosphates	Carbohydrates

Glucose 6-phosphate	g6p	Sugar 6 phosphates	Sugar phosphates	Carbohydrates
D-Galactose	gal	Deoxy sugars	Monosaccharides	Carbohydrates
D-Galactarate	galct-D	Dicarboxylic acids	Carboxylic acids	Organic acids
D-Galactonate	galctn-D	Dicarboxylic acids	Carboxylic acids	Organic acids
D-Galacturonate	galur	Dicarboxylic acids	Carboxylic acids	Organic acids
D-Glucosamine 6-phosphate	gam	Amino sugars	Monosaccharides	Carbohydrates
D-Glucosamine	gam6p	Sugar 6 phosphates	Sugar phosphates	Carbohydrates
D-Glucose	glc-D	Aldoses	Monosaccharides	Carbohydrates
D-Gluconate	glcn	Sugar acids	Monosaccharides	Carbohydrates
D-Glucarate	glcr	Dicarboxylic acids	Carboxylic acids	Organic acids
D-Glucuronate	glcur	Dicarboxylic acids	Carboxylic acids	Organic acids
L-Glutamine	gln-L	Common amino acids	Amino acids	Peptides
L-Glutamate	glu-L	Common amino acids	Amino acids	Peptides
Glutarate	glutar	Dicarboxylic acids	Carboxylic acids	Organic acids
Glyoxylate	glx	2-Oxocarboxylic acids	Carboxylic acids	Organic acids
Glycine-L-Asparagine	gly-asn-L	Dipeptides	Amino acids	Peptides
Glycine-L-Aspartic acid	gly-asp-L	Dipeptides	Amino acids	Peptides
L-Cysteine-Glycine	gly-cys-L	Dipeptides	Amino acids	Peptides
Glycine-L-Glutamine	gly-gln-L	Dipeptides	Amino acids	Peptides
Glycine-L-Glutamate	gly-glu-L	Dipeptides	Amino acids	Peptides
Glycine-L-Methionine	gly-met-L	Dipeptides	Amino acids	Peptides
Glycine-L-Phenylalanine	gly-phe-L	Dipeptides	Amino acids	Peptides
Glycine-L-Proline	gly-pro-L	Dipeptides	Amino acids	Peptides
Glycine-L-Tyrosine	gly-tyr-L	Dipeptides	Amino acids	Peptides
Glycine	gly	Common amino acids	Amino acids	Peptides
Glycerol 3-phosphate	glyc	Sugar alcohols	Monosaccharides	Carbohydrates
Glycerol	glyc3p	Sugar 3 phosphates	Sugar phosphates	Carbohydrates
Glycolate	glyclt	Dicarboxylic acids	Carboxylic acids	Organic acids
L-Histidine	his-L	Common amino acids	Amino acids	Peptides
L-Isoleucine	ile-L	Common amino acids	Amino acids	Peptides
Inosine	ins	Ribonucleosides	Nucleosides	Nucleic acids
D-Lactate	lac-D	Hydroxycarboxylic acids	Carboxylic acids	Organic acids
L-Lactate	lac-L	Hydroxycarboxylic acids	Carboxylic acids	Organic acids
Lactose	lcts	Disaccharides	Disaccharides	Carbohydrates
L-Leucine	leu-L	Common amino acids	Amino acids	Peptides
L-Lysine	lys-L	Common amino acids	Amino acids	Peptides
L-Lyxose	lyx-L	Aldoses	Monosaccharides	Carbohydrates
Alpha Methyl D glucoside	madg	Carbohydrate derivatives	Glycans	Carbohydrates

D-Malate	mal-D	Hydroxycarboxylic acids	Carboxylic acids	Organic acids
L-Malate	mal-L	Hydroxycarboxylic acids	Carboxylic acids	Organic acids
Maltose	malt	Disaccharides	Disaccharides	Carbohydrates
Maltotriose	malttr	Polysaccharides	Polysaccharides	Carbohydrates
D-Mannose	man	Aldoses	Monosaccharides	Carbohydrates
Beta Methylglucoside	mbdg	Carbohydrate derivatives	Glycans	Carbohydrates
Melibiose	melib	Disaccharides	Disaccharides	Carbohydrates
L-Methionine-L-Alanine	met-L-ala-L	Dipeptides	Amino acids	Peptides
L-Methionine	met-L	Common amino acids	Amino acids	Peptides
Mannitol	mnl	Sugar alcohols	Monosaccharides	Carbohydrates
Ornithine	orn-L	Other amino acids	Amino acids	Peptides
Phenylacetic acid	pac	Monocarboxylic acids	Carboxylic acids	Organic acids
Phenethylamine	peamn	Biogenic amines	Amines	Peptides
L-Phenylalanine	phe-L	Common amino acids	Amino acids	Peptides
Pimelate	pime	Dicarboxylic acids	Carboxylic acids	Organic acids
Propionate	ppa	Monocarboxylic acids	Carboxylic acids	Organic acids
L-Proline	pro-L	Common amino acids	Amino acids	Peptides
Putrescine	ptrc	Biogenic amines	Amines	Peptides
Pyruvate	pyr	2-Oxocarboxylic acids	Carboxylic acids	Organic acids
D-Ribose	rib-D	Aldoses	Monosaccharides	Carbohydrates
L-Rhamnose	rmn	Deoxy sugars	Monosaccharides	Carbohydrates
D-Sorbitol	sbt-D	Sugar alcohols	Monosaccharides	Carbohydrates
L-Serine	ser-L	Common amino acids	Amino acids	Peptides
Spermidine	spmd	Biogenic amines	Amines	Peptides
Succinate	succ	Dicarboxylic acids	Carboxylic acids	Organic acids
Sucrose	sucr	Disaccharides	Disaccharides	Carbohydrates
D-tartrate	tartr-D	Dicarboxylic acids	Carboxylic acids	Organic acids
L-tartrate	tartr-L	Dicarboxylic acids	Carboxylic acids	Organic acids
Tricarballylate	tcb	Tricarboxylic acids	Carboxylic acids	Organic acids
L-Threonine	thr-L	Common amino acids	Amino acids	Peptides
Thymidine	thymd	Deoxyribonucleosides	Nucleosides	Nucleic acids
Trehalose	tre	Disaccharides	Disaccharides	Carbohydrates
L-Tryptophan	trp-L	Common amino acids	Amino acids	Peptides
Tyramine	tym	Biogenic amines	Amines	Peptides
L-Tyrosine	tyr-L	Common amino acids	Amino acids	Peptides
Uridine	uri	Ribonucleosides	Nucleosides	Nucleic acids
L-Valine	val-L	Common amino acids	Amino acids	Peptides
D-Xylose	xyl-D	Aldoses	Monosaccharides	Carbohydrates
Xylitol	xylt	Sugar alcohols	Monosaccharides	Carbohydrates

Supplementary Table 2: Minimal medium components

Metabolite Name
p-Aminobenzoic acid
Biotin
Calcium
Vitamin B12 (cobalamin)
Choline
Chlorine
Cobalt
Copper
Iron (II)
Iron (III)
Folate
Hydrogen
Water
Potassium
Lipoate (Thiocitic acid)
Magnesium
Manganese
Molybdate
Sodium
Nicotinamide
Ammonium
Nickel
Nitrate
Oxygen (only in oxic conditions)
Phosphate
R-Pantothenate
Pyridoxal
Riboflavin
Selenite
Sulfate
Thiamine
Zinc

Supplementary Table 3: Dynamical modeling parameters and initial conditions

	Variable	Value
Parameters	$\mu_{\max}$	[0-1] hr^{-1}
	D	0.2 hr^{-1}
	k_c	0.04 $g{*}L^{-1}$
	I_{m}	4 $g{*}L^{-1}$
	K_m	0.023 $g_{nutrient*}g_{cells}^{-1}$
	$k_{\tilde{m}}$	10 hr^{-1}
	$K_{\tilde{m},s}$	0.025 $g_{nutrient*}g_{cells}^{-1}$
	$k_{s,\tilde{m}}$	0.04 $g{*}L^{-1}$
Initial Conditions	s_1	0.1 $g{*}L^{-1}$
	s_2	0.1 $g{*}L^{-1}$
	m_α	4 $g{*}L^{-1}$
	m_β	4 $g{*}L^{-1}$
	$\tilde{m}_1$	0 $g{*}L^{-1}$
	$\tilde{m}_2$	0 $g{*}L^{-1}$

Supplementary Table 4: Comparison to known secretion/interaction profiles

**Predicted secretion/exchange phenomenon**	**Observed secretion/exchange phenomenon**	**References**
Secretion of central carbon intermediates and other metabolites in *E. coli* and *S. cerevisiae* in appropriate medium conditions	*E. coli* and *S. cerevisiae* observed to secrete Glucose-6-phosphate, Fructose-6-phosphate, Fructose-1,6-bisphosphate, Glyceraldehyde-3-phosphate, Phosphoenolpyruvate, Pyruvate, Acetyl-CoA, Citrate, Isocitrate, α-Ketoglutarate, Fumarate, Malate, and Glyoxylate. *E. coli* observed to secrete Acetate, Oroate, and Uracil. *S. cerevisiae* observed to secrete ethanol. All in varying environmental conditions.	2<br>3
Secretion of metabolites by soil-associate microbes, such as *Bacillus subtilis*	85 unique metabolites, including nucleotides, organic acids, vitamins, carbohydrates, and amino acids were observed to be secreted in a soil microbial community.	4
Exchange of intermediate organic acids by anaerobic organisms	Transfer of electrons between organisms through diffusible chemical compounds (e.g. organic acids, amino acids)	5<br>6
Secretion of amino acids in *E. coli* and *S. cerevisiae* in appropriate medium conditions	*E. coli* and *S. cerevisiae* observed to secrete Alanine, Valine, Aspartate, Homoserine, Methionine, Tryptophan, Tyrosine, Proline, Serine, Glycine, and Arginine. *E. coli* observed to secrete Leucine, Isoleucine, Threonine, Phenylalanine, Glutamine, and Histidine. *S. cerevisiae* observed to secrete Glutamate and Lysine. All in varying environmental conditions.	2<br>7<br>8<br>9
Synechocystis sp. PCC6803 takes up ammonium as a nitrogen source in most cases of metabolic exchange	Various cyanobacteria are known to preferentially use ammonium as a nitrogen source when available.	10<br>11
Exchange of amino acids between bacteria and yeast	Yeast species provide amino acids to lactic acid bacteria	12<br>13

Supplementary Table 5: List of metabolites predicted under growth maximization objective (maxGro) but not under one or more of the following objective functions: growth minimization (minGro), ATP maximization (maxATP), and ATP minimization (minATP). N_S is defined as the total number of simulations in which a metabolite was secreted.

Metabolite	N_S, maxGro	N_S, minGro	N_S, maxATP	N_S, minATP	Secreting organisms
1,5-Diaminopentane	26	0	26	0	*M. extorquens* (no O_2)
Agmatine	249	0	644	0	*S. enterica* (no O_2)
L-arginine	276	0	377	0	*P. aeruginosa* (no O_2)<br>*S. cerevisiae* (no O_2)
5'-Deoxyadenosine	3148	0	0	0	*S. cerevisiae* (no O_2)
Sulfite	185	0	169	0	*P. aeruginosa* (no O_2)<br>*S. oneidensis* (with O_2)
Sulfate	1	0	2	0	*S. cerevisiae* (with O_2)
Spermidine	13	0	13	0	*S. cerevisiae* (with O_2)
L-sorbose	1391	0	1391	0	*P. aeruginosa* (no O_2)<br>*S. cerevisiae* (no O_2)<br>*S. enterica* (no O_2)
L-tryptophan	13	0	13	0	*S. cerevisiae* (no O_2)
Thiosulfate	2151	0	2253	0	*E. coli* (no O_2)<br>*K. pneumoniae* (no O_2, with O_2)<br>*S. boydii* (no O_2)<br>*S. enterica* (no O_2)<br>*S. oneidensis* (no O_2)

Supplementary Table 6: Metabolite exchange frequencies for habitat-specific simulations. N_E is defined as the total number of simulations in which a metabolite was exchanged. In total, there were 72,026, 94,269, and 120,662 simulations in which there was at least one metabolite exchanged in aquatic, soil, and gut habitats respectively.

Aquatic (With O_2)		Soil (With O_2)		Gut (No O_2)	
**Metabolite**	N_E	**Metabolite**	N_E	**Metabolite**	N_E
Acetate	39744	Acetate	42489	L-Serine	67913
Carbon dioxide	19457	Carbon dioxide	31366	L-Glutamine	67912
Ammonium	18743	Oxygen	26000	L-Cysteine	67831
Succinate	12580	Ammonium	21199	L-Leucine	67830
Urea	11242	Formate	21186	L-Methionine	67828
Iron (II)	10240	Urea	18607	Phosphate	67357
L-Alanine	8374	Succinate	18157	Ammonium	44862
Formate	7775	Pyruvate	8651	L-Threonine	44839
Pyruvate	7658	L-Alanine	8391	Nicotinate	44809
Hydrogen	6927	D-Lactate	6787	Maltohexaose	34541
D-Lactate	6636	L-Malate	6779	L-Arginine	34040
L-Malate	6025	L-Tryptophan	5685	L-Valine	33930
L-Tryptophan	4680	Phosphate	5572	L-Isoleucine	33918
Phosphate	4219	Iron (II)	4534	L-Tryptophan	33890
Citrate	2475	Hydrogen	3549	L-Proline	32664
Glycolate	1980	Glycerol	3354	L-Alanine	28111
L-Arginine	1344	Xanthine	2124	L-Tyrosine	28110
L-Glutamate	1300	Guanine	2063	Carbon dioxide	27539
Glycine	1241	L-Arginine	1513	Ethanol	24641
Xanthine	1085	L-Glutamate	1483	Orotate	24180
Fumarate	1027	Glycine	1353	Formate	20453
Guanine	941	Thymine	1093	L-Lactate	12109
Adenine	602	2-Oxoglutarate	1064	Folate	11472
L-Proline	593	Allantoin	902	Succinate	9226
Allantoin	573	Adenine	899	Glycine	9179
Indole	529	Hypoxanthine	835	Acetate	8144
Thymine	497	L-Proline	696	Hydrogen	7829
Hypoxanthine	424	Ethanol	546	Nitrite	4008
Glycerol	420	Fumarate	545	Glycolate	2284
2-Oxoglutarate	308	Indole	527	Iron (II)	779
Ethanol	303	5-Methylthio-D-ribose	413	Adenine	744
L-Valine	282	L-Valine	329	Isocitrate	577
L-Lactate	244	L-Lactate	312	Urea	470
L-Ornithine	213	L-Cysteine	304	Hypoxanthine	464
Uracil	213	L-Ornithine	250	Xanthine	406
L-Cysteine	133	Adenosine 3,5-bisphosph.	237	Uracil	400
Putrescine	125	Methanol	225	Allantoin	242
(S)-Propane-1,2-diol	112	Thiosulfate	185	Fumarate	150
Biotin	91	Uracil	151	(S)-Propane-1,2-diol	132
L-Aspartate	77	(S)-Propane-1,2-diol	112	Butyrate	113
Hexadecanoate	23	L Sorbose	105	Propanal	104
(R)-Glycerate	15	L-Aspartate	101	Hexanoate	103
Choline	11	Xylitol	101	Putrescine	99
D-Alanine	10	Putrescine	100	Thymine	76
L-Isoleucine	5	Biotin	91	Agmatine	43
		Hydrogen peroxide	91	L-Ornithine	32
		Hexadecanoate	36	Propionate	16
		Choline	33	L-Asparagine	5

		Phenylacetaldehyde	27	Guanine	4
		Nitrate	26		
		Citrate	7		
		dTTP	6		
		L-Isoleucine	6		
		(R)-Propane-1,2-diol	3		
		Propionate	3		

Supplementary Table 7: Carbon sources listed according to cooperativity index clustering for oxic and anoxic simulations. List order follows clustering in Supplementary Figure 8 (down y-axis, across x-axis).

With O_2	No O_2
D-Glucose	D-Glucose
Pyruvate	D-Sorbitol
L-Glutamine	Sucrose
L-Serine	Glycerol 3-phosphate
L-Glutamate	Inosine
L-Alanine	D-Ribose
L-Lactate	D-Glucosamine
Glycine	Adenosine
L-Arginine	Mannitol
L-Malate	D-Mannose
L-Ornithine	Trehalose
L-Aspartic acid	D-Xylose
2-Oxoglutarate	L-Serine
D-Sorbitol	D-Galactose
Mannitol	Maltose
Acetate	Pyruvate
Citrate	Melibiose
Fumarate	L-Glutamate
L-Asparagine	Citrate
Succinate	Glycerol
Sucrose	L-Glutamine
Uridine	Uridine
D-Alanine	L-Malate
Adenosine	N-Acetyl-D-glucosamine
Inosine	L-Aspartic acid
Glycerol 3-phosphate	D-Alanine
D-Ribose	L-Alanine
L-Proline	L-Histidine
L-Threonine	Glycine
N-Acetyl-D-glucosamine	D-Glucosamine 6-phosphate
Ethanol	L-Lactate
Putrescine	D-Glucose 1-phosphate
Trehalose	Glucose 6-phosphate
D-Galactose	D-Fructose 6-phosphate
D-Xylose	2-Oxoglutarate
D-Glucosamine	L-Ornithine
D-Mannose	L-Arginine
D-Lactate	D-Glucarate
Glycerol	Succinate
Melibiose	L-Proline
Maltose	Thymidine
D-Glucarate	D-Galacturonate
Glycolate	D-Malate
D-Glucuronate	L-Threonine
D-Malate	Fumarate
Maltotriose	L-Rhamnose
Thymidine	Ethanol
D-Galacturonate	L-Fucose
Propionate	D-Glucuronate
D-Glucose 1-phosphate	Maltotriose
D-Glucosamine 6-phosphate	L-Asparagine

D-Fructose 6-phosphate	Lactose
Glucose 6-phosphate	L-Lyxose
L-Histidine	D-Galactonate
Formate	Glycolate
Lactose	N-Acetyl-D-mannosamine
L-Rhamnose	D-Galactarate
L-Fucose	Putrescine
D-Galactarate	L-Isoleucine
Cellobiose	Propionate
D-Galactonate	D-Lactate
L-Isoleucine	L-Leucine
N-Acetyl-D-mannosamine	L-Valine
L-Valine	Cellobiose
L-Cysteine	Formate
L-Lyxose	Glutarate
L-Alanine-L-Glutamate	Xylitol
L-Leucine	Acetate
Glycine-L-Proline	L-Cysteine
L-Tryptophan	L-Tryptophan
L-tartrate	L-Alanine-L-Glutamate
D-tartrate	L-tartrate
L-Lysine	D-tartrate
Xylitol	Acetoacetate
L-Phenylalanine	Tricarballylate
Spermidine	Butyrate
Glutarate	L-Methionine
L-Tyrosine	L-Lysine
Dextrin	L-Phenylalanine
Acetoacetate	L-Tyrosine
Tricarballylate	Glycine-L-Proline
Glyoxylate	Glyoxylate
Alpha Methyl D glucoside	Dextrin
Beta Methylglucoside	Phenylacetic acid
Phenethylamine	Spermidine
L-Methionine	D-Fructose
Butyrate	D-Gluconate
Deoxyadenosine	L-Alanine-L-Asparagine
L-Methionine-L-Alanine	L-Alanine-L-Threonine
Phenylacetic acid	L-Alanine-L-Leucine
Pimelate	L-Alanine-L-Histidine
D-Gluconate	Glycine-L-Phenylalanine
Glycine-L-Tyrosine	Glycine-L-Tyrosine
D-Fructose	L-Alanine-L-Glutamine
Glycine-L-Methionine	Deoxyadenosine
Glycine-L-Glutamate	Glycine-L-Methionine
Glycine-L-Glutamine	Glycine-L-Asparagine
Glycine-L-Cysteine	Glycine-L-Aspartic acid
Glycine-L-Aspartic acid	Glycine-L-Cysteine
Glycine-L-Phenylalanine	Glycine-L-Glutamine
Glycine-L-Asparagine	Glycine-L-Glutamate
L-Alanine-L-Asparagine	L-Methionine-L-Alanine
L-Alanine-L-Glutamine	Phenethylamine
L-Alanine-L-Histidine	Pimelate
L-Alanine-L-Leucine	Tyramine
L-Alanine-L-Threonine	1,3-Propanediol
1,3-Propanediol	Beta Methylglucoside
Tyramine	Alpha Methyl D glucoside

SUPPLEMENTARY REFERENCES

1. King, Z. A. *et al.* BiGG Models: A platform for integrating, standardizing and sharing genome-scale models. *Nucleic Acids Res.* **44,** D515–D522 (2016).
2. Paczia, N. *et al.* Extensive exometabolome analysis reveals extended overflow metabolism in various microorganisms. *Microb. Cell Fact.* **11,** 1–14 (2012).
3. Smith, M. G., Des Etages, S. G. & Snyder, M. Microbial synergy via an ethanol-triggered pathway. *Mol. Cell. Biol.* **24,** 3874–84 (2004).
4. Swenson, T. L., Karaoz, U., Swenson, J. M., Bowen, B. P. & Northen, T. R. Linking soil biology and chemistry in biological soil crust using isolate exometabolomics. *Nat. Commun.* **9,** (2018).
5. Morris, B. E. L., Henneberger, R., Huber, H. & Moissl-Eichinger, C. Microbial syntrophy: Interaction for the common good. *FEMS Microbiol. Rev.* **37,** 384–406 (2013).
6. Embree, M., Liu, J. K., Al-Bassam, M. M. & Zengler, K. Networks of energetic and metabolic interactions define dynamics in microbial communities. *Proc. Natl. Acad. Sci.* **112,** 15450–15455 (2015).
7. Airich, L. G. *et al.* Membrane topology analysis of the Escherichia coli aromatic amino acid efflux protein YddG. *J. Mol. Microbiol. Biotechnol.* **19,** 189–97 (2010).
8. Dassler, T., Maier, T., Winterhalter, C. & Bock, A. Identification of a major facilitator protein from Escherichia coli involved in efflux of metabolites of the cysteine pathway. *Mol. Microbiol.* **36,** 1101–1112 (2000).
9. Velasco, I., Tenreiro, S., Calderon, I. L. & André, B. Saccharomyces cerevisiae Aqr1 is an internal-membrane transporter involved in excretion of amino acids. *Eukaryot. Cell* **3,** 1492–503 (2004).
10. Lindell, D. & Post, A. F. Ecological Aspects of *ntcA* Gene Expression and Its Use as an Indicator of the Nitrogen Status of Marine *Synechococcus* spp. *Appl. Environ. Microbiol.* **67,** 3340–3349 (2001).
11. Glibert, P. M. & Ray, R. T. Different patterns of growth and nitrogen uptake in two clones of marineSynechococcus spp. *Mar. Biol.* **107,** 273–280 (1990).
12. Stadie, J., Gulitz, A., Ehrmann, M. A. & Vogel, R. F. Metabolic activity and symbiotic interactions of lactic acid bacteria and yeasts isolated from water kefir. *Food Microbiol.* **35,** 92–98 (2013).
13. Ponomarova, O. *et al.* Yeast Creates a Niche for Symbiotic Lactic Acid Bacteria through Nitrogen Overflow. *Cell Syst.* **5,** 345–357.e6 (2017).
