# Supplementary material for: Costless metabolic secretions as drivers of interspecies interactions in microbial ecosystems
Source: Nat Commun. 2019 Jan 9;10:103. doi: 10.1038/s41467-018-07946-9 (PMC6327061; doi:10.1038/s41467-018-07946-9)
Supplement: Supplementary file 3 — Description of Additional Supplementary Files [file 41467_2018_7946_MOESM3_ESM.pdf]

Description of Additional Supplementary Files

File Name: Supplementary Data 1
Description: List of genome-scale metabolic models used in pairwise analyses

File Name: Supplementary Data 2
Description: List of metabolites secreted in dynamic FBA simulations, with organisms under differing substrate concentrations.
